# Involvement of Src kinases and PLCγ2 in clot retraction

**DOI:** 10.1016/j.thromres.2006.09.003

**Published:** 2007

**Authors:** Katsue Suzuki-Inoue, Craig E. Hughes, Osamu Inoue, Makoto Kaneko, Olga Cuyun-Lira, Toshiro Takafuta, Steve P. Watson, Yukio Ozaki

**Affiliations:** a Department of Clinical and Laboratory Medicine, Faculty of Medicine, Yamanashi University, 1110 Shimokato, Chuo, Yamanashi, 409–3898, Japan; b Centre for Cardiovascular Sciences, Institute of Biomedical Research, Division of Medical Sciences, The Medical School, University of Birmingham, Edgbaston, Birmingham B15 2TT, UK; c Department of Clinical Laboratory, The University of Tokyo Hospital, Tokyo, 113–8655, Japan; d Department of Hematology and Clinical Immunology, Nishi-kobe Medical Center, Kobe, 651-2273, Japan

**Keywords:** ACD, acid/citrate/dextrose, mAb, monoclonal antibody, SLP-76, SH2-containing leucocyte phosphoprotein of 76 kDa, PLCγ2, phospholipase Cγ2, PP2, 4-amino-5-(4-chlorophenyl)-7-(*t*-butyl)pyrazolo-d-3,4-pyrimidine, PRP, platelet-rich plasma, TBS-T, Tris-buffered saline/Tween 20., Blood platelets, Clot retraction, Integrin α_IIb_β_3_, PLCγ2, Src kinases, Outside-in signal

## Abstract

The integrin α_IIb_β_3_ plays a critical role in mediating clot retraction by platelets which is important *in vivo* in consolidating thrombus formation. Actin–myosin interaction is essential for clot retraction. In the present study, we demonstrate that the structurally distinct Src kinase inhibitors, PP2 and PD173952, significantly reduced the rate of clot retraction, but did not prevent it reaching completion. This effect was accompanied by abolition of α_IIb_β_3_-dependent protein tyrosine phosphorylation, including PLCγ2. A role for PLCγ2 in mediating clot retraction was demonstrated using PLCγ2-deficient murine platelets. Furthermore, platelet adhesion to fibrinogen leads to MLC phosphorylation through a pathway that is inhibited by PP2 and by the PLC inhibitor, U73122. These results demonstrate a partial role for Src kinase-dependent activation of PLCγ2 and MLC phosphorylation in mediating clot retraction downstream of integrin α_IIb_β_3_.

## Introduction

Activation of integrin α_IIb_β_3_ by ‘inside-out’ signals leads to binding of fibrinogen and platelet aggregation. In turn, clustering of α_IIb_β_3_ as a consequence of engagement of α_IIb_β_3_ by fibrinogen mediates ‘outside-in’ signals that stimulate tyrosine phosphorylation of β_3_-integrin tail [1] and activation of a Src-based signalling cascade [2] that involves Syk [3,4], SLP-76 [5] and PLCγ2 [6,7]. Binding of Syk to the α_IIb_β_3_-tail is inhibited by tyrosine phosphorylation of the β_3_-integrin tail [8], suggesting that these two integrin-dependent signalling cascades are distinct. Activation of PLCγ2 downstream of activation of Src kinases has been shown to be essential for platelet spreading (lamellipodia formation) on fibrinogen [2,6,7], whereas the role of tyrosine phosphorylation of the β_3_-integrin tail in this response is not known.

It is well established that integrin α_IIb_β_3_ plays a critical role in regulating clot retraction by platelets, which serves to consolidate thrombus formation. Clot retraction is mediated by the interaction of fibrin and the actin cytoskeleton via integrin α_IIb_β_3_, together with activation of the platelet contractile apparatus [9]. Tyrosine phosphorylation of the β_3_-integrin tail has been shown to play a role in mediating clot retraction, but is not essential. Platelets from a mutant mouse in which the two conserved tyrosines in the β_3_-integrin tail, positions 747 and 759, have been mutated to phenylalanine (diY-F mutant) exhibit impaired clot retraction *in vitro*, in line with recurrent bleeding *in vivo* following tail excision [10]. Significantly, these observations are consistent with a reported role for tyrosine kinases in cytoskeletal attachment to α_IIb_β_3_ and retraction of fibrin polymers [11]. Paradoxically, however, it has been reported that the time course of clot retraction parallels that of protein tyrosine dephosphorylation [12]. The explanation for these contrasting observations is unclear.

In the present study, we have investigated the contribution of α_IIb_β_3_-dependent regulation of Src kinases and PLCγ2 in the process of clot retraction in platelets. The results reveal a partial, but non-essential role for Src kinases and PLCγ2 in mediating clot retraction in platelets. The results support a model in which outside-in signalling through integrin α_IIb_β_3_ to PLCγ2 contributes to the regulation of the contractile apparatus that underlies clot retraction.

## Materials and methods

### Antibodies and reagents

Anti-phospho-MLC monoclonal antibody (mAb) or anti-MLC polyclonal Ab (pAb) were kindly donated by Drs. Koichiro Fukuda and Yasuharu Sasaki (Frontier 21 Project, Life Science Center, Asahi Chemical, Shizuoka, Japan). PD173952 was a gift from Pfizer (Ann Arbor, Michigan, USA) [13]. Myosin II inhibitor, blebbistatin(-), its inactive enantiomer blebbistatin(+), Rho kinase inhibitor Y-27632, Src kinase inhibitor PP2, and its inactive control PP3 were from Calbiochem (CA, USA). Human fibrinogen and thrombin were obtained from Sigma (MO, USA). Integrin α_IIb_β_3_ blocking peptide GRGDS was from Peptide Institute (Osaka, Japan). PLCγ2-deficient mice were obtained as previously described [14]. Anti-PLCγ2 antibody was obtained from Santa Cruz Biotechnology (CA, USA).

### Preparation of human and mouse platelets

Venus blood from drug-free volunteers was taken into 10% sodium citrate. Platelet-rich plasma was obtained after centrifugation at 1100 rpm for 12 min. 15% acid–citrate–dextrose and 250 ng/ml of prostaglandin I_2_ were added, and the platelet-rich plasma (PRP) was centrifuged at 2500 rpm for 10 min. Human platelets were resuspended in modified Tyrodes buffer (137 mM NaCl, 11.9 mM NaHCO_3_, 0.4 mM Na_2_HPO_4_, 2.7 mM KCl, 1.1 mM MgCl_2_, 5.6 mM glucose, pH 7.3), washed again, and resuspended at a cell density of 5 × 10^8^/ml. Murine blood (approximately 1 ml) was drawn from CO_2_ terminally-narcosed mice by portal vein puncture and taken into 100 μl of 4% sodium citrate. The citrated blood was added to 0.7 vol. of modified Tyrodes buffer. PRP was obtained by centrifugation at 200g for 5 min. To obtain murine washed platelets, murine blood was drawn into 100 μl of acid citrate dextrose and PRP was obtained by centrifugation at 200 *g* for 5 min. Plasma was removed by centrifugation at 1000 *g* for 10 min in the presence of 1 μg/ml of PGI_2_. In both PRP and washed platelets, cell densities were adjusted to 3 × 10^8^/ml with Tyrodes buffer.

### Clot retraction assay of human and murine platelets

For human washed platelets, clot retraction studies were performed at 20 °C in an air incubator in an aggregometer tube. Assays were started by adding 250 μl of 2 U/ml thrombin to 250 μl of platelets (5 × 10^8^/ml) in the presence of 2 mg/ml fibrinogen and 2 mM CaCl_2_ (final concentrations: 2.5 × 10^8^/ml of platelets, 1 U/ml of thrombin, 1 mg/ml of fibrinogen, 1 mM CaCl_2_). For murine diluted-PRP (400 μl), assays were performed at 37 °C in an aggregometer tube containing thrombin and CaCl_2_ to give the final concentrations: 3 × 10^8^/ml of platelets, 10 U/ml of thrombin, 2 mg/ml fibrinogen and 2 mM CaCl_2_. These conditions were chosen so that clot retraction proceeds with a similar time course to that seen with human platelets. Where indicated, human platelets or murine diluted-PRP were preincubated with inhibitors or vehicle solution for 60 min at room temperature or for 10 min at 37 °C, respectively. Clot retraction was recorded by digital camera, Cyber-shot (Sony, Tokyo, Japan) and by measurement of the volume of clear fluid that could be removed [10].

### Platelet aggregation

Washed human platelets (5 × 10^8^/ml) were preincubated with 50 μM PP3, 50 μM PP2, 80 μM blebbistatin(-), 80 μM blebbistatin(+), DMSO, or 20 μM Y-27632 for 5 min at 37 °C. Platelets were stimulated with 1 U/ml of thrombin and platelet aggregation was monitored in an aggregometer AA100 (Kowa Co. Ltd., Tokyo, Japan) for 5 min at 37 °C.

### Western blotting and immunoprecipitation studies

For measurement of tyrosine phosphorylation, clot retraction was terminated by addition of 2× lysis buffer [15]. Samples were sonicated for 3 periods of 15 s each and insoluble debris removed by centrifugation at 15,000 *g* for 10 min. PLCγ2 was precipitated by anti-PLCγ2 antibody as described [6,15]. Samples were also taken and solubilized by addition of 4× SDS sample buffer for analysis of total protein tyrosine phosphorylation. Platelet proteins were separated by SDS-PAGE and blotted with anti-phosphotyrosine antibody (4G10) to detect protein tyrosine phosphorylation as described previously [6,15].

### MLC phosphorylation during platelet spreading on fibrinogen-coated surfaces

Plastic dishes for cell culture (6 cm) were coated with 0.5 ml fibrinogen (500 μg/ml) overnight at 4 °C. After removing unbound fibrinogen, dishes were washed with phosphate-buffered saline and blocked with 1% BSA for 2 h at room temperature. Human or murine washed platelets (0.4 ml at 3 × 10^8^/ml), pretreated with 10 μM indomethacin and 3 U/ml of apyrase, were seeded on BSA- or fibrinogen-coated surfaces in the presence of the inhibitors. Bound and unbound platelets were solubilized by 4× Laemmli sample buffer, followed by immediate sonication for three periods of 15 s. Platelet proteins were separated by SDS-PAGE on 15% gels and electrotransferred. Phospho-MLC or total MLC were blotted using anti-phospho-MLC mAb or anti-MLC pAb [16].

### Statistics

The data are expressed as the mean ± SE. Data were analyzed with a one or two-tailed, non-parametric Mann–Whitney *U*-test. *p* value less than 0.05 was considered statistically significant.

## Results

### Critical role of Src kinases in supporting clot retraction

**Figure 1:**
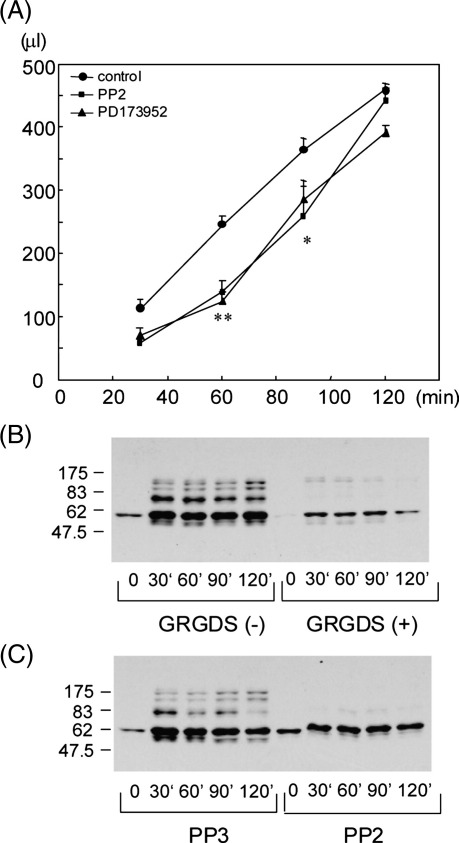
The Src kinase inhibitors and α_IIb_β_3_ blockade by GRGDS peptide reduced time course of clot retraction and inhibited protein tyrosine phosphorylation during clot retraction. (A) Human washed platelets (5 × 10^8^/ml) were preincubated with DMSO, 50 μM PP2, 40 μM PD173952. Clot retraction assays were started by adding 250 μl of 2 U/ml thrombin to 250 μl of platelets in the presence of 2 mg/ml fibrinogen and 2 mM CaCl_2_ (final concentrations: 2.5 × 10^8^/ml of platelets, 1 U/ml of thrombin, 1 mg/ml of fibrinogen, 1 mM CaCl_2_). The volume of remaining fluid was measured to assay the degree of clot retraction. The volume was expressed as mean ± SE (*n* = 5–18 from 2–5 experiments). Results were analyzed using unpaired Student's *t*-test. One asterisk denotes *p* < 0.05 and two denote *p* < 0.005 between control and PP2/PD173952. (B) Human washed platelets in the presence or absence of 1 mM GRGDS peptide were stimulated by 250 μl of 2 U/ml thrombin as described. Reactions were terminated by addition of 2× lysis buffer. Samples were sonicated 3 periods of 15 s and insoluble debris removed by centrifugation at 15,000 *g* for 10 min. The supernatant was solubilized by addition of 4× SDS sample buffer. The platelet proteins were separated by SDS-PAGE and blotted with anti-phosphotyrosine antibody (4G10). (C) Human washed platelets (5 × 10^8^/ml) were preincubated with 50 μM PP3 or PP2 before starting assays of clot retraction. Protein tyrosine phosphorylation was analyzed as described above.

Clot retraction was analyzed in washed platelets in the presence of fibrinogen (1 mg/ml) and thrombin (1 unit/ml) by measuring the volume of fluid that could be withdrawn from the platelet suspension. Experiments were carried out at 20 °C to reduce the time course of response. Clot retraction increased steadily up to 120 min, at which time between 80 and 90% of the original suspension could be recovered (Fig. 1A). Clot retraction was slowed in the presence of concentrations of the structurally distinct Src kinase inhibitors, PP2 and PD173952, which are known to cause maximal inhibition of Src kinase in platelets (Fig. 1A) [14,17,18]. The response recovered to within 20% of the control by 120 min (Fig. 1A). These results demonstrate a contributory but not essential role for Src kinases in clot retraction.

The time course of protein tyrosine phosphorylation during clot retraction was analyzed. A marked increase in tyrosine phosphorylation of several proteins in the whole cell lysate was observed which was maintained for up to 120 min (Fig 1B). The apparent contradiction with a recent report of transient phosphorylation during clot retraction [12] may reflect the lower temperature used in the present study. Tyrosine phosphorylation was markedly inhibited in the presence of the α_IIb_β_3_-blocking peptide, GRGDS (Fig. 1B) and the structurally distinct Src kinase inhibitors, PP2 (Fig. 1C) and PD173952 (not shown), but not by the inactive analogue of PP2, PP3 (Fig. 1C). These results illustrate that the major increase in tyrosine phosphorylation is mediated downstream of α_IIb_β_3_-dependent activation of a Src kinase-dependent pathway, consistent with previous reports [2].

### PLCγ2 contributes to clot retraction

**Figure 2:**
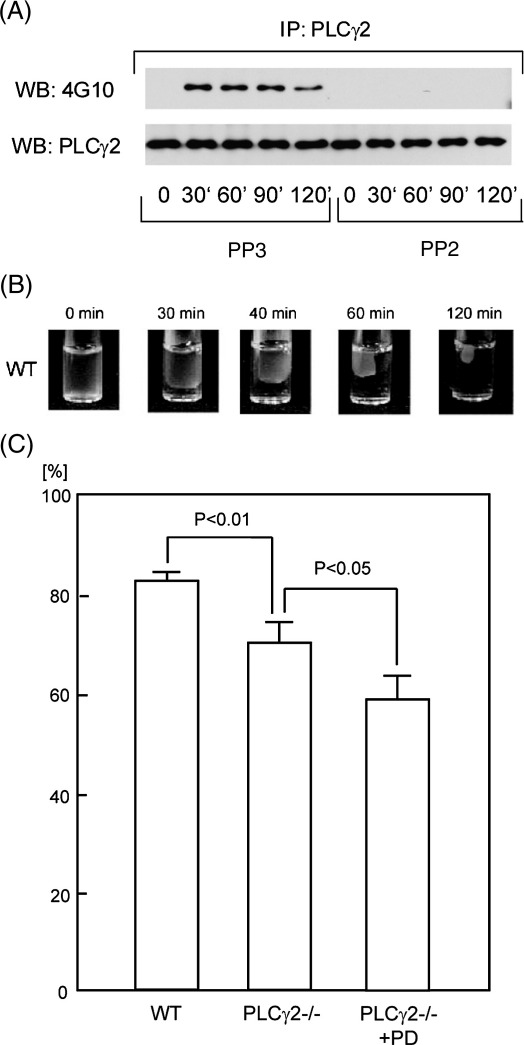
A role for PLCγ2 in clot retraction. (A) Human washed platelets (5 × 10^8^/ml) were preincubated with 50 μM PP3 or PP2 before starting assays of clot retraction. Clot retraction was stimulated as described in the legend of Fig. 1 and reactions were terminated by addition of 2× lysis buffer. Samples were sonicated 3 periods of 15 s and insoluble debris removed by centrifugation at 15,000 *g* for 10 min. PLCγ2 was immunoprecipitated using the supernatant. The immunoprecipitates were separated by SDS-PAGE and blotted with anti-phosphotyrosine antibody (4G10) or anti-PLCγ2. (B, C) Murine diluted-PRP (400 μl, 3 × 10^8^/ml) from wild type mice or PLCγ2-deficient mice was added 10 U/ml of thrombin and 2 mM CaCl_2_. Where indicated, PLCγ2-deficient platelets were preincubated with 40 μM PD173952 before starting clot retraction assay. Clot retraction was recorded by digital camera (B) and the volume of remaining fluid was measured at 120 min to assay the degree of clot retraction (C). The results were shown as a percentage of fluid that could be removed. The percentage of fluid was expressed as mean ± SE (*n* = 6–9 from 3 experiments). Data were analyzed with a one-tailed Mann–Whitney *U*-test. *P* value less than 0.05 was considered statistically significant.

Adhesion of platelets to a fibrinogen monolayer induces tyrosine phosphorylation of PLCγ2 through a Src kinase-dependent pathway [6,7]. Consistent with this, thrombin stimulated a marked increase in tyrosine phosphorylation of PLCγ2, which was dependent on engagement of integrin α_IIb_β_3_ as demonstrated using the α_IIb_β_3_ antagonist, GRGDS peptide (not shown). Tyrosine phosphorylation of PLCγ2 declined slightly by 120 min and was inhibited in the presence of PP2 (Fig. 2A).

The role of PLCγ2 in the process of clot retraction was investigated using PLCγ2-deficient murine platelets. Conditions were chosen such that thrombin stimulated a similar time course of clot retraction in mouse platelet-rich plasma to that seen in human platelets. Clot retraction was detectable by 30 min and increased steadily up to 120 min, at which time approximately 80% of the original suspension could be recovered as clear fluid (Fig. 2B). The degree of clot retraction was reduced by approximately 15% in the absence of PLCγ2 (*p* = 0.0028; Fig. 2C). Interestingly, the degree of clot retraction was further reduced by over 25% when PLCγ2-deficient platelets were pretreated with Src kinase inhibitor PD173952, which is freely available in plasma [13] (Fig. 2C). This level of inhibition was significantly greater than that seen in the absence of PLCγ2 (*p* = 0.033), but was not significantly different from that induced by PD173952 on its own (data not shown). This indicates the presence of an additional Src kinase-dependent mechanism of clot retraction, which could be mediated, for example, through activation of PLCγ1 [14] or downstream of tyrosine phosphorylation of the integrin β_3_-tail [19]. These results therefore show that, under these conditions, the role of Src kinases in clot retraction is mediated in part through inhibition of PLCγ2.

### Regulation of MLC phosphorylation events in supporting clot retraction

**Figure 3:**
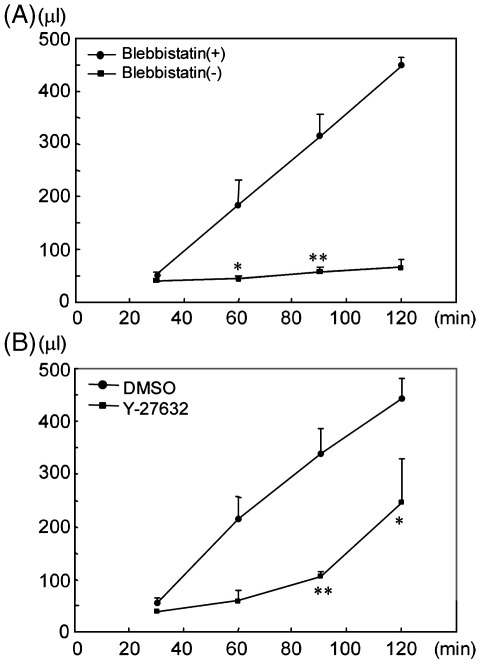
Myosin inhibitor, blebbistatin(-) and Rho kinase inhibitor, Y-27632 inhibited clot retraction. Human washed platelets (5 × 10^8^/ml) were preincubated with 80 μM blebbistatin(-), 80 μM blebbistatin(+) (A), DMSO, or 20 μM Y-27632 (B). Clot retraction assays were started by adding 250 μl of 2 U/ml thrombin to 250 μl of platelets in the presence of 2 mg/ml fibrinogen and 2 mM CaCl_2_ (final concentrations: 2.5 × 10^8^/ml of platelets, 1 U/ml of thrombin, 1 mg/ml of fibrinogen, 1 mM CaCl_2_). The volume of remaining fluid was measured to assay the degree of clot retraction. The volume was expressed as mean ± SE (*n* = 4–7 from 2 experiments). Results were analyzed using unpaired Student's *t*-test. One asterisk denotes *p* < 0.05 and two denote *p* < 0.005 between the controls and the inhibitors.

It is well established that actin–myosin contraction plays an essential role in mediating clot retraction. In line with this, clot retraction in human platelets was inhibited completely in the presence of a selective inhibitor of nonmuscle myosin II, blebbistatin(-) [20] (Fig. 3A), whereas the inactive enantiomer blebbistatin(+) had no effect on clot retraction (Fig. 3A). Blebbistatin(-) had no effect on thrombin-induced platelet aggregation (not shown), illustrating that its inhibitory effect was not due to inhibition of “inside-out” activation of α_IIb_β_3_.

Actin–myosin cross-bridge formation is regulated by phosphorylation of MLC, which can be brought about by Ca^2+^-dependent activation of MLC kinase and through Rho kinase-dependent inhibition of MLC phosphatase. Thrombin regulates both of these pathways through activation of PAR_1_ and PAR_4_ receptors, each of which couple to G_q_ and G_12/13_ heterotrimeric G proteins [21]. A role for Rho kinase in mediating clot retraction is illustrated by the partial inhibition of response in the presence of the Rho kinase inhibitor, Y-27632, which reduced clot retraction by 30–40% at 120 min (Fig. 3B). In comparison, the same concentration of Y-27632 had no effect on aggregation (not shown), demonstrating that this effect was not mediated by inhibition of thrombin-induced activation of α_IIb_β_3_. A previous report that inhibition of Rho by C3-exozyme has no effect on clot retraction [22], may reflect the incomplete inhibition of Rho activity that was observed in this study.

**Figure 4:**
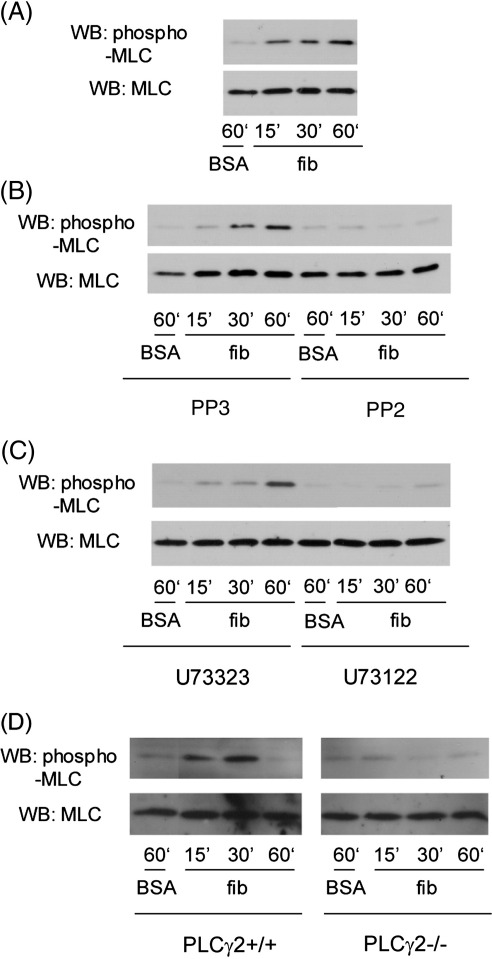
α_IIb_β_3_ outside-in regulation of MLC phosphorylation was inhibited by Src inhibitor or PLC inhibitor. 0.4 ml of human washed platelets at 3 × 10^8^/ml pretreated with 10 μM indomethacin and 3 U/ml of apyrase followed by being incubated without (A) or with 50 μM PP2, PP3 (B), 10 μM U73122, or U73323 (C), and then they were seeded on the surfaces coated with BSA or fibrinogen (fib) for indicated times. Bound and unbound platelets were solubilized by 4× Laemmli sample buffer, followed by immediate sonication for three periods of 5 s. Platelet proteins were separated by SDS-PAGE on 15% gels, electrotransferred, and then phospho-MLC or total MLC were blotted using anti-phospho-MLC mAb or anti-MLC pAb. (D) 0.4 ml of washed platelets at 3 × 10^8^/ml from PLCγ2+/+ and PLCγ2-/- mice were pretreated with 10 μM indomethacin and 3 U/ml of apyrase and then they were seeded on the surfaces coated with BSA or fibrinogen (fib) for indicated times. The following procedure is the same as that for human platelets. The data are representatives of at least two experiments.

Potentially, clot retraction could also be regulated through activation of PLCβ by thrombin and/or PLCγ2 by α_IIb_β_3_, thereby leading to Ca^2+^ elevation and activation of MLC kinase. In consideration of this, we investigated whether outside-in signals by α_IIb_β_3_ activate MLC phosphorylation in the absence of thrombin. To address this, we seeded washed, human platelets on a fibrinogen-coated surface in the absence of thrombin. Indomethacin and apyrase were included in these studies to prevent stimulation of phosphorylation through release of thromboxane A_2_ and ADP. Immobilized fibrinogen stimulated weak MLC phosphorylation (Fig. 4A), which was inhibited in the presence of the Src kinase inhibitors, PP2 (Fig. 4B) and PD173952 (not shown), and by the phospholipase inhibitor, U73122 (Fig. 4C). The inactive enantiomers of PP2 and U73122, namely PP3 and U73323 respectively, had no effect (Fig. 4B and C). Moreover, MLC phosphorylation in platelets seeded on immobilized fibrinogen is inhibited in PLCγ2-deficient mice (Fig. 4D). These findings confirm that α_IIb_β_3_ stimulates MLC phosphorylation through a pathway that is partially dependent on Src kinases and PLCγ2.

## Discussion

**Figure 5:**
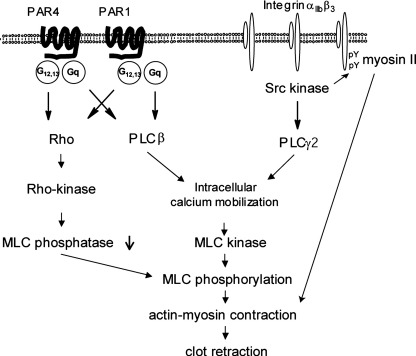
Multiple mechanisms of regulation of the contractile apparatus underlie clot retraction. Thrombin receptors are able to regulate clot retraction through activation of PLCβ and Rho kinase. α_IIb_β_3_ outside-in signalling is required for optimal clot retraction through bifurcating signals, namely activation of PLCγ2 and phosphorylation of the diY motif, which combine to mediate clot retraction. This interaction could occur by recruitment of myosin to the phosphorylated diY motif in combination of activation of MLC kinase downstream of PLCγ2.

The present study demonstrates that α_IIb_β_3_ outside-in signalling is required for optimal clot retraction in thrombin-stimulated platelets through a pathway that is partially dependent on Src kinases and PLCγ2. Significantly, however, this α_IIb_β_3_-regulated cascade is not essential for completion of clot retraction, most likely because clot retraction is regulated through a number of additional pathways, including activation of PLCβ and Rho kinase, and α_IIb_β_3_-regulation of PLCγ1, which is expressed in low level in mouse platelets [14]. A schematic summarizing these results is shown in Fig. 5. As expected, however, clot retraction is absolutely dependent on myosin contractility, as demonstrated using the inhibitor of myosin II, blebbistatin. The present results therefore indicate that clot retraction in thrombin-stimulated platelets is regulated through multiple pathways, including thrombin-dependent activation of PLCβ and Rho kinase, which activate MLC kinase and inhibit MLC phosphatase, respectively, and α_IIb_β_3_-regulation of PLCγ2, mediated downstream of Src kinase activation (Fig. 5).

A role for α_IIb_β_3_ outside-in signalling via PLCγ2 in clot retraction should be considered in the context of a previous study on a mutant mouse in which the two conserved tyrosines in the β_3_-integrin tail, at positions 747 and 759, were mutated to phenylalanine residues. Significantly, dual mutation of these residues also results in a partial impairment in clot retraction, the importance of which is illustrated by an increase in rebleeding following incision of the tail [10]. Phosphorylation of tyrosine 747 and 759 in the β_3_ tail is also blocked by inhibition of Src kinase activation [19], but is independent of activation of PLCγ2 by Syk as demonstrated using Syk-deficient murine platelets (Hughes, Hughan and Watson, unpublished observation). These results therefore suggest a model in which bifurcating signals from α_IIb_β_3_, namely activation of PLCγ2 and phosphorylation of the diY motif, combine to mediate clot retraction. This interaction could occur, for example, by recruitment of myosin to the phosphorylated diY motif in combination of activation of MLC kinase downstream of PLCγ2 [23] (Fig. 5). The observation that an increased inhibition of clot retraction is seen in the presence of the Src kinase inhibitor PD173952 relative to that observed in the absence of PLCγ2 is consistent with this possibility, although the difference could also be due to the presence of PLCγ1 in murine platelets [14].

The demonstration of the presence of multiple routes of clot retraction in platelets is consistent with the physiological importance of this phenomenon. Multiple routes of regulation of MLC phosphorylation may have evolved to ensure rapid activation of clot retraction at sites of damage to the vasculature in order to withstand the high shear forces found within small arteries and arterioles. The physiological importance of outside-in signalling by α_IIb_β_3_ in clot retraction is illustrated by the rebleeding that occurs in the Di-YF mouse [10]. The present results imply a significant contribution of Src kinase-dependent regulation of PLCγ2 downstream of α_IIb_β_3_ in this process (Fig. 5).

## References

[1] Law D.A., Nannizzi-Alaimo L., Phillips D.R. (1996). Outside-in integrin signal transduction. Alpha IIb beta 3-(GP IIb IIIa) tyrosine phosphorylation induced by platelet aggregation. J Biol Chem.

[2] Obergfell A., Eto K., Mocsai A., Buensuceso C., Moores S.L., Brugge J.S. (2002). Coordinate interactions of Csk, Src, and Syk kinases with [alpha]IIb[beta]3 initiate integrin signaling to the cytoskeleton. J Cell Biol.

[3] Clark E.A., Shattil S.J., Ginsberg M.H., Bolen J., Brugge J.S. (1994). Regulation of the protein tyrosine kinase pp72syk by platelet agonists and the integrin alpha IIb beta 3. J Biol Chem.

[4] Gao J., Zoller K.E., Ginsberg M.H., Brugge J.S., Shattil S.J. (1997). Regulation of the pp72syk protein tyrosine kinase by platelet integrin alpha IIb beta 3. EMBO J.

[5] Obergfell A., Judd B.A., del Pozo M.A., Schwartz M.A., Koretzky G.A., Shattil S.J. (2001). The molecular adapter SLP-76 relays signals from platelet integrin alphaIIbbeta3 to the actin cytoskeleton. J Biol Chem.

[6] Wonerow P., Pearce A.C., Vaux D.J., Watson S.P. (2003). A critical role for phospholipase Cgamma 2 in alpha IIbeta 3-mediated platelet spreading. J Biol Chem.

[7] Goncalves I., Hughan S.C., Schoenwaelder S.M., Yap C.L., Yuan Y., Jackson S.P. (2003). Integrin alpha IIbbeta 3-dependent calcium signals regulate platelet–fibrinogen interactions under flow: involvement of PLCgamma 2. J Biol Chem.

[8] Woodside D.G., Obergfell A., Talapatra A., Calderwood D.A., Shattil S.J., Ginsberg M.H. (2002). The N-terminal SH2 domains of Syk and ZAP-70 mediate phosphotyrosine-independent binding to integrin beta cytoplasmic domains. J Biol Chem.

[9] Shattil S.J., Kashiwagi H., Pampori N. (1998). Integrin signaling: the platelet paradigm. Blood.

[10] Law D.A., DeGuzman F.R., Heiser P., Ministri-Madrid K., Killeen N., Phillips D.R. (1999). Integrin cytoplasmic tyrosine motif is required for outside-in alphaIIbbeta3 signalling and platelet function. Nature.

[11] Schoenwaelder S.M., Jackson S.P., Yuan Y., Teasdale M.S., Salem H.H., Mitchell C.A. (1994). Tyrosine kinases regulate the cytoskeletal attachment of integrin alpha IIb beta 3 (platelet glycoprotein IIb/IIIa) and the cellular retraction of fibrin polymers. J Biol Chem.

[12] Osdoit S., Rosa J.P. (2001). Fibrin clot retraction by human platelets correlates with alpha(IIb)beta(3) integrin-dependent protein tyrosine dephosphorylation. J Biol Chem.

[13] Marshall S.J., Senis Y.A., Auger J.M., Feil R., Hofmann F., Salmon G. (2003). GPIb-dependent platelet activation is dependent on Src kinases but not MAP kinase or cGMP-dependent kinase. Blood.

[14] Suzuki-Inoue K., Inoue O., Frampton J., Watson S.P. (2003). Murine GPVI stimulates weak integrin activation in PLC{gamma}2-/- platelets: involvement of PLC{gamma}1 and PI 3-kinase. Blood.

[15] Inoue O., Suzuki-Inoue K., Dean W.L., Frampton J., Watson S.P. (2003). Integrin alpha2beta1 mediates outside-in regulation of platelet spreading on collagen through activation of Src kinases and PLCgamma2. J Cell Biol.

[16] Fukuda K., Ozaki Y., Satoh K., Kume S., Tawata M., Onaya T. (1997). Phosphorylation of myosin light chain in resting platelets from NIDDM patients is enhanced: correlation with spontaneous aggregation. Diabetes.

[17] Rosado J.A., Graves D., Sage S.O. (2000). Tyrosine kinases activate store-mediated Ca2^+^; entry in human platelets through the reorganization of the actin cytoskeleton. Biochem J.

[18] Ohmori T., Yatomi Y., Okamoto H., Miura Y., Rile G., Satoh K. (2001). G(i)-mediated Cas tyrosine phosphorylation in vascular endothelial cells stimulated with sphingosine 1-phosphate: possible involvement in cell motility enhancement in cooperation with Rho-mediated pathways. J Biol Chem.

[19] Phillips D.R., Nannizzi-Alaimo L., Prasad K.S. (2001). Beta3 tyrosine phosphorylation in alphaIIbbeta3 (platelet membrane GP IIb-IIIa) outside-in integrin signaling. Thromb Haemost.

[20] Straight A.F., Cheung A., Limouze J., Chen I., Westwood N.J., Sellers J.R. (2003). Dissecting temporal and spatial control of cytokinesis with a myosin II Inhibitor. Science.

[21] Coughlin S.R. (2000). Thrombin signalling and protease-activated receptors. Nature.

[22] Leng L., Kashiwagi H., Ren X.D., Shattil S.J. (1998). RhoA and the function of platelet integrin alphaIIbbeta3. Blood.

[23] Jenkins A.L., Nannizzi-Alaimo L., Silver D., Sellers J.R., Ginsberg M.H., Law D.A. (1998). Tyrosine phosphorylation of the beta3 cytoplasmic domain mediates integrin–cytoskeletal interactions. J Biol Chem.

